# Prospective Evaluation of Two Cohorts of Non-Operatively Treated Patients with Displaced vs. Minimally and Non-Displaced Distal Radius Fractures

**DOI:** 10.3390/jcm12052076

**Published:** 2023-03-06

**Authors:** Rikke Thorninger, Daniel Wæver, Michael Tjørnild, Martin Lind, Jan Duedal Rölfing

**Affiliations:** 1 Department of Orthopaedics, Regional Hospital Randers, Skovlyvej 15, 8930 Randers, Denmark; 2 Department of Clinical Medicine, HEALTH, Aarhus University, Palle Juul-Jensens Boulevard 82, 8200 Aarhus, Denmark; 3 Department of Orthopaedics, Aarhus University Hospital, Palle Juul-Jensens Boulevard 99, J801, 8200 Aarhus, Denmark

**Keywords:** distal radius fracture, fracture, non-operative treatment, conservative treatment, complications, patient-reported outcome measures, QuickDASH, PRWHE, NRS, osteoporosis, aging

## Abstract

Background: Distal radius fractures (DRFs) in the elderly are common. Recently, the efficacy of operative treatment of displaced DRFs in patients above 65 years of age has been questioned and it has been suggested that non-operative treatment should be the gold standard. However, the complications and functional outcome of displaced vs. minimally and non-displaced DRFs in the elderly has not been evaluated yet. The aim of the present study was to compare non-operatively treated displaced DRFs vs. minimally and non-displaced DRFs in terms of complications, PROMs, grip strength and range of motion (ROM) after 2 weeks, 5 weeks, 6 months and 12 months. Methods: We used a prospective cohort study that compared patients with displaced DRFs (n = 50), i.e., >10 degrees of dorsal angulation after two reduction attempts, with patients with minimally or non-displaced DRFs after reduction. Both cohorts received the same treatment of 5 weeks of dorsal plaster casting. Complications and functional outcomes (quick disabilities of the arm, shoulder and hand (QuickDASH), patient-rated wrist/hand evaluation (PRWHE), grip strength and EQ-5D scores) were assessed after 5 weeks, 6 months and 12 months post-injury. The protocol of the VOLCON RCT and present observational study has been published (PMC6599306; clinicaltrials.gov: NCT03716661). Results: One year after 5 weeks of dorsal below-elbow casting of low-energy DRFs in patients ≥ 65 years old, we found a complication rate of 6.3% (3/48) in minimally or non-displaced DRFs and 16.6% (7/42) in displaced DRFs (*p* = 0.18). However, no statistically significant difference was observed in functional outcomes in terms of QuickDASH, pain, ROM, grip strength or EQ-5D scores. Discussion: In patients above 65 years of age, non-operative treatment, i.e., closed reduction and dorsal casting for 5 weeks, yielded similar complication rates and functional outcomes after 1 year regardless of whether the initial fracture was non-displaced/minimally displaced or still displaced after closed reduction. While the initial closed reduction should still be attempted in order to restore the anatomy, failure to achieve the stipulated radiological criteria may not be as important as we thought in terms of complications and functional outcome.

## 1. Introduction

Distal radius fractures (DRFs) have a bimodal distribution with high-energy fractures in the young and low-energy fractures in the elderly and post-menopausal women in particular [1]. Due to the growing elderly population, the socio-economic burden of DRFs is increasing [2].

Over the last two decades, there has been a trend towards open reduction and internal fixation (ORIF) with volar locking plates (VLP) of displaced DRFs. Indications for surgery are stipulated in the clinical guidelines [3]. Minimally or non-displaced DRFs are commonly treated non-operatively with a cast.

Recent evidence questions the benefit of surgical treatment of DRFs in the elderly [4,5,6,7,8,9]. At 12 months follow-up there was no difference in patient-related outcome measures (PROM), i.e., QuickDASH (quick disabilities of the arm, shoulder and hand) and PRWHE (patient-rated wrist/hand evaluation). Consequently, the latest guidelines from the American Academy of Orthopedic Surgery have adopted this evidence [10]. However, the British guidelines state that “In patients 65 years of age or older, non-operative treatment can be considered as a primary treatment for dorsally displaced DRF unless there is significant deformity or neurological compromise” [11]. Counselling of patients regarding the expected rehabilitation, complications and functional outcome of non-operative treatment of displaced as well as minimally and non-displaced DRFs lacks evidence.

**Figure 1:**
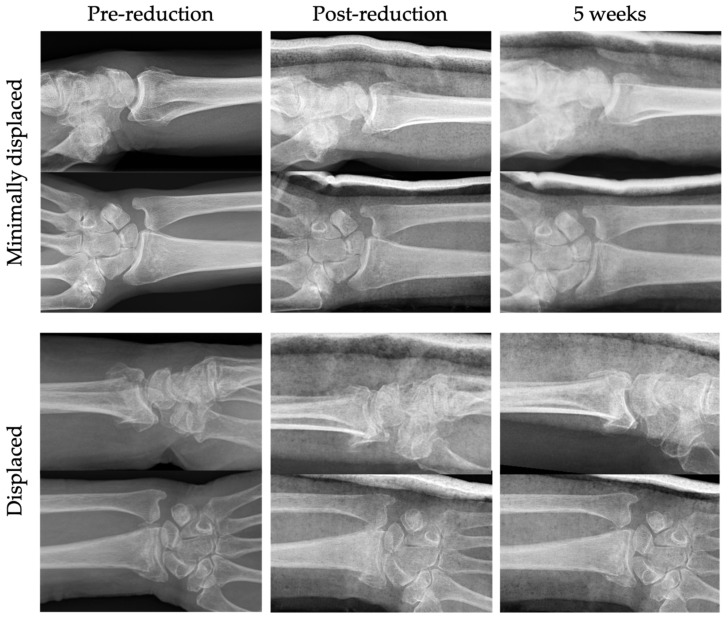
Examples of radiographs of the two non-operatively treated cohorts: minimally displaced distal radius fracture (DRF) and displaced DRF after two attempts of fluoroscopic guided closed reduction. Pre-reduction, post-reduction and 5 week follow up.

The aim of the present study was to compare non-operatively treated displaced DRFs vs. minimally/non-displaced DRFs (Figure 1) in terms of complications, PROMs, grip strength and range of motion (ROM) after 2 and 5 weeks, as well as after 6 and 12 months. To the best of our knowledge, no studies have investigated this relationship before.

## 2. Materials and Methods

In this prospective observational study, we evaluated two cohorts of displaced DRFs vs. minimally/non-displaced DRFs in patients above 65 years of age treated with a dorsal cast for 5 weeks. The study protocol for both the VOLCON randomized controlled trial (RCT) and the present observational prospective cohort study has been published (PMC6599306; clinicaltrials.gov: NCT03716661) [12]. All patients gave written consent to participate in the study and could withdraw their consent at any time. STROBE reporting guidelines for observational studies were followed.

### 2.1. Cohorts

**Figure 2:**
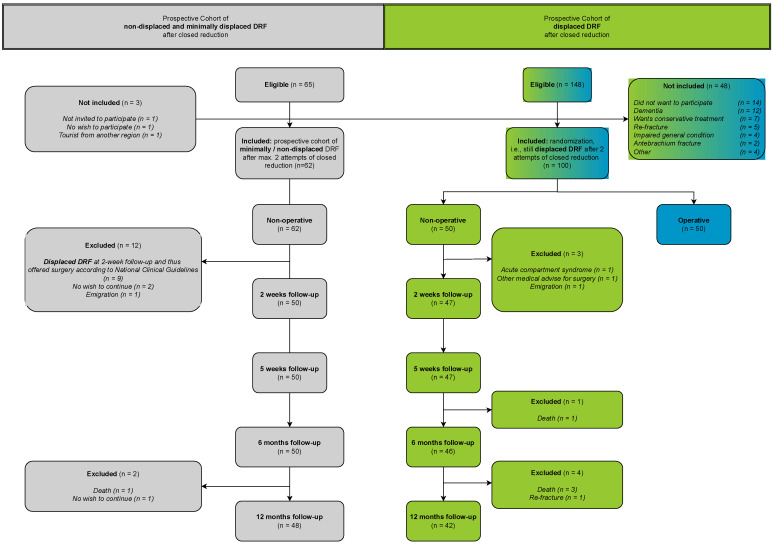
CONSORT flow diagram of the study including cohort 1 (minimally and non-displaced distal radius fractures) and cohort 2 (“non-operative” arm of the VOLCON randomized controlled trial). Numbers of eligible, included and excluded patients, as well as reasons for exclusion, are given. The eligible patients of the non-operative arm of the RCT had to consent to participate in the RCT in order to be included in the present study (green).

All ≥ 65-year-old patients presenting with a low-energy DRF at the emergency department (ED), Regional Hospital Randers, Denmark were eligible (Figure 2). Physicians had a maximum of two attempts of closed reduction and casting under fluoroscopic guidance using a hematoma block in order to achieve an acceptable reduction according to the radiological criteria of the National Clinical Guideline [3]. After each reduction attempt under fluoroscopic guidance, standard radiographs were taken at the Department of Radiology. These radiographs were assessed for acceptable reduction. If the radiological measurements were not acceptable, another attempt of reduction was performed. Patients were chosen above the age of 65 as it reflects the retirement age in Denmark. The Danish National Clinical Guidelines for surgery in displaced DRFs reflect the guidelines of the American Academy of Orthopedic Surgeons (AAOS) and British Society for Surgery of the Hand (BSSH) [10,11].

Cohort 1: defined as minimally or non-displaced or displaced DRF with acceptable reduction according to the radiological criteria of the national clinical guideline (n1 = 50) (Figure 2). 

Cohort 2: defined as a displaced DRF with unacceptable reduction according to the National Clinical Guidelines after 2 attempts of closed reduction (n2 = 50). In the published RCT, patients from cohort 2 were randomized to either operative or non-operative treatment, i.e., the same treatment as cohort 1 comprised of 5 weeks of a dorsal, below-elbow cast and a single instruction by a hand therapist at the time of cast removal.

The two cohorts were prospectively evaluated regarding primary and secondary outcomes as described below at day 0 recalling the pre-injury state, 2 weeks, 5 weeks, 6 months and 12 months. Patients with high-energy fractures, open fractures, former ipsilateral fractures, concomitant fractures and patients unable to provide written consent were excluded.

### 2.2. Primary Outcome Measure

The primary outcome was the number of patients suffering from one or more of the complications listed below compared between the two cohorts.

The complication rate was prospectively assessed at day 0 recalling the pre-injury state and after 2 weeks, 5 weeks, 6 months and 12 months after the injury. The patient answered standardized questions from the investigators (R.T. and D.W.) who were aware of the purpose of the study and the group allocation as they also included the patient on the same day. In order to assess the day 0 complication rate correctly, we took the following measures as stated in the published protocol: “Patients will report complications at the given timepoints by answering a questionnaire stating either yes/no and a free-text explanation. If the patient states any complications, a member of the research group will qualify the answer and fill in the free text. However, a “yes” can only be qualified and shall never be erased if the physician does not agree with the patient’s opinion or explanation” [12]. Patients were able to state additional comments, if the complication was not on the predefined list. Moreover, we reviewed the patients’ medical journal in order to check for additional complications, which the patient may have forgotten/failed to self-report. Complications were defined according to the published protocol [12]:

- Carpal tunnel syndrome and chronic regional pain syndrome;
- Unspecific sensory disturbances;
- Flexor tendon rupture and irritation;
- Extensor tendon rupture and irritation;
- Infection: superficial/deep;
- Vascular compromised (capillary refill ≥2 s).

Regarding infection, superficial infection may occur due to pressure ulcers of the cast. Deep infection in closed, non-operatively treated DRFs is extremely rare, but it may arise from a hematogenous spread of bacteria.

### 2.3. Secondary Outcome Measures

Quick disabilities of the arm, shoulder and hand (QuickDASH) questionnaire was used to assess the patient-reported functional outcome [13,14,15]. The minimally functional clinical important difference (MCID) is a 16 to 20-point QuickDASH difference in accordance with the recommendations of the developers [13,16,17].

The patient-rated wrist/hand evaluation (PRWHE) was also applied and constitutes a self-reported assessment of 5 items on pain, 10 items on function and 2 optional items on appearance [18]. The MCID for PRWHE was set to 10 points [19].

Active range of motion (ROM) of the wrist (flexion, extension, pronation, supination, radial deviation, and ulnar deviation) was measured with a goniometer by an independent, blinded observer during the follow-up period. Blinding consisted of wearing stockings on the wrists in order to conceal scars and deformities. The primary reason for concealing the distal radius with a stocking was to blind the trained nurses, who performed the ROM assessment because the stocking may mask a mild deformity. Secondly, the nurses evaluated both non-operatively treated patients of the present study in the same time period as operatively treated patients of the VOLCON RCT (blue boxes of the CONSORT flow diagram, Figure 2) [9]. The nurses were, thus, completely unaware of the group allocation and performed treatment. The ROM of the contralateral uninjured wrist served as reference. Only healthy contralateral wrists were included in the analysis.

Grip strength was measured using an electronic hand dynamometer (EH101 CAMRY). Grip strength is given as the mean of three measurements on each side [20,21]. The MCID of grip strength was 6.5 kg [22].

EuroQol-5D (EQ-5D-3L) was used to estimate quality of life using national population weights.

### 2.4. Statistical Analysis

The complication rate was analyzed using Fisher’s exact test of the accumulated complication rate after 12 months. Double counting was avoided in patients with multiple complications as only one complication was accounted for per patient.

Secondary outcome measures were analyzed using mixed-effects analysis with Sidak’s multiple-comparison test. According to our sample size calculation of the RCT, 50 patients per treatment arm provide 80% statistical power at a 5% alpha level, assuming a difference of 20% in complication rate between operatively and non-operatively treated patients. Prism 9 for macOS was used for statistical analysis and graphs.

The study was performed in accordance with the Declaration of Helsinki, prospectively registered at clinicaltrials.gov (NCT03716661) and approved by the Danish Scientific Ethical Committee (1–10–72-420-17) [12].

## 3. Results

Figure 2 presents the number of eligible, included and excluded patients. The baseline demographics of the two cohorts and the AO/OTA fracture classification are given in Table 1.

### 3.1. Primary Outcome: Complications

In the minimally or non-displaced DRF group, 3 out of 48 patients experienced complications within the first year. The complication rate was, thus, 6.3% after 12 months; two patients complained about unspecific sensory disturbances and one patient complained about a lack of strength compared with the preoperative state as well as swelling. These complaints could be objectively qualified with a low grip strength measurement and were, thus, rated as complications. Moreover, two patients complained about pain during activity after 12 months. The latter two subjective statements were not rated as complications. The complication rate was 16.6% (7 out of 42 patients) in the displaced DRF group consisting of two superficial wounds without signs of infection at cast removal after 5 weeks, two carpal tunnel syndromes treated with surgical decompression after 5 weeks and 11 months, respectively, and three unspecific sensory disturbances at 12-month follow-up. This difference of 3/48 vs. 7/42 patients was not statistically significant (*p* = 0.18, Fisher’s exact test).

### 3.2. Secondary Outcomes: Functional Outcome

**Figure 3:**
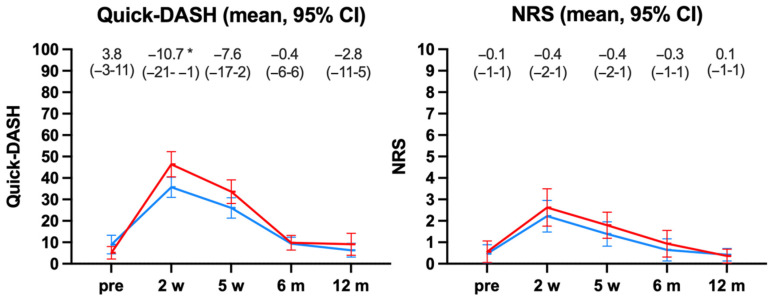
QuickDASH and NRS (pain) of the displaced (red) and minimally/non-displaced (blue). non-operatively treated DRFs. Mean differences (95% CI) between the groups at the different timepoints and statistical significance * *p* < 0.05.

QuickDASH and NRS were comparable at baseline (recalled pre-injury state), 6 and 12 months. The mean difference in QuickDASH of −10.7 (from −21 to −1) between displaced vs. minimally/non-displaced DRF was statistically significant after 2 weeks (Figure 3), but of borderline clinical relevance as the MCID was 16–20 points [17].

The mean PRWHE of displaced DRF patients was 12.6 (8.7–16.5) after 6 months and 8.0 (3.6–12.4) after 12 months, while for minimally or non-displaced DRF patients it was 13.5 (9.0–18.0) after 6 months and 8.7 (3.6–13.7) after 12 months. Accordingly, the mixed-effects model showed a time dependency (*p* = 0.01) but not a treatment dependency of PRWHE (*p* = 0.79).

**Figure 4:**
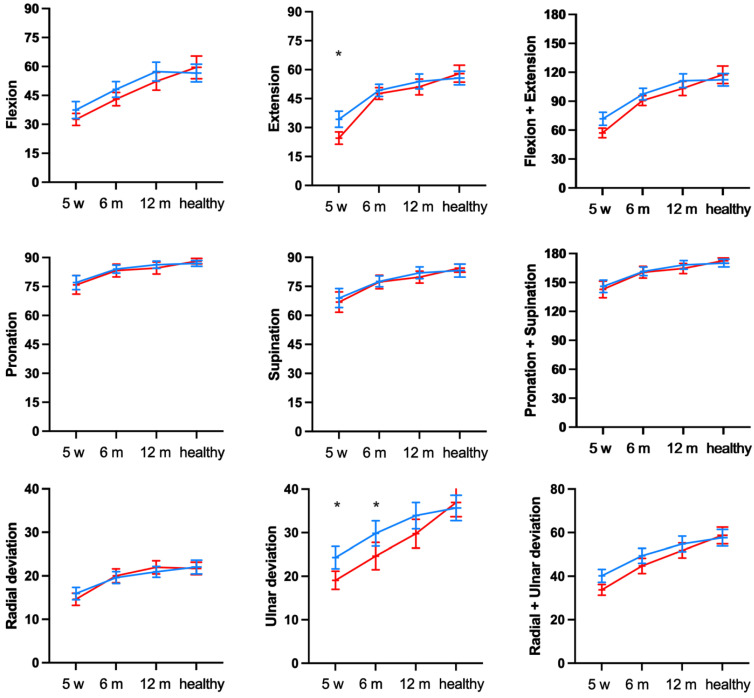
Mean range of motion of wrists with displaced (red) and minimally/non-displaced (blue) DRF at 5 weeks (w), 6 and 12 months (m) compared with the uninjured/healthy side. Error bars represent 95% confidence intervals. * *p* < 0.05.

The ROM of both groups was significantly impaired after cast removal at 5 weeks and after 6 months compared with the uninjured wrist (Figure 4; *p* < 0.05). The ulnar deviation (Figure 4, lower-middle panel) was the only direction of movement where the graphs did not align, and statistical analysis revealed impairment in the displaced compared with the minimally or non-displaced group after both 5 weeks and 6 months (Figure 4, lower middle panel).

For the remaining directions of movement, the overlaying graphs and statistical analyses highlighted that the improvements over time were similar in both cohorts and no statistically significant differences were observed compared with the uninjured side after 12 months (*p* > 0.05).

The mean grip strength was low in both cohorts, i.e., 18.8 kg (14.1–23.6) for minimally or non-displaced DRFs and 16.6 kg (11.8–21.4) for displaced DRFs after 12 months. The mean difference in grip strength between the groups was 0.5 (−2.2–3.2) after 6 months and 1.2 (−4.0–1.6) after 12 months.

EQ5D-3L indices of patients with minimally or non-displaced DRF improved from 0.87 (95% CI 0.84–0.90, range 0.68–1.00) at 6 months to 0.93 (95% CI 0.90–0.96, range 0.71–1.00 after 12 months post-injury. In the displaced DRF group the corresponding values were 0.79 (95% CI 0.72–0.86, range 0.28–1.00) and 0.84 (95% CI 0.76–0.93, range 0.14–1.00).

## 4. Discussion

One year after 5 weeks of dorsal below-elbow casting of low-energy DRFs in patients ≥ 65 years old, we found a complication rate of 6.3% (3/48) in minimally or non-displaced DRFs and 16.6% (7/42) in displaced DRFs (*p* = 0.18). However, no statistically significant difference was observed in the functional outcome in terms of the QuickDASH, pain, ROM, grip strength, or EQ-5D scores.

Complications of the present study were pre-defined according to the published RCT protocol. While making an effort to streamline the interpretation of complications, we did not grade the severity. Notably, the two carpal tunnel syndromes occurred in the displaced group leading to decompression surgery of the medial nerve, which resolved the symptoms at the latest follow-up. Our finding of 2/90 (2.2%) patients with carpal tunnel syndrome within the first 12 months was low/comparable with reports in the literature, stating an incidence of 6.3% in the first 6 months in 1198 out of 18,766 non-operatively treated DRF patients [23]. While hypothetical, it seems logical that the displacement of bony fragments in a displaced DRF may cause swelling and compression of the median nerve. In corroboration with this statement, we did not observe classic symptoms of carpal tunnel syndrome in any minimally or non-displaced DRFs, in which the bony anatomy was better restored during closed reduction. The increased swelling may also hypothetically have led to the two cases of superficial wounds noted upon cast removal at 5 weeks. Moreover, one patient returned to the ED for a cast exchange after swelling had subsided. This event was not accounted for as a complication.

The definition of complications after a DRF is not arbitrary; taken together, the complications in both groups were mild and temporary. We have previously reported that the unspecific sensory disturbances after a DRF change over time and may, thus, not necessarily be a lasting complication and at final follow-up there was no motoric compromise and the sensory disturbance did not follow an anatomical innervation pattern [9,24]. However, all of these patients were informed about the symptoms and findings of medial and ulnar nerve compression as a precautious measure at the latest follow-up. Moreover, one patient mentioned diminished grip strength as a complication. It may be a matter of debate if this is a true complication, but we chose to adhere to the protocol, where we stated: “If the patient states any complications, a member of the research group will qualify the answer and fill in the free text. However, a YES can only be qualified and shall never be erased if the physician does not agree with the patient’s opinion or explanation”.

Regarding grip strength, one may speculate if the difference between the cohorts may be related to the frailty of the patients, for example, due to osteoporosis and sarcopenia [25,26]. Contrary to our a priori expectations, in the present study 3/50 displaced DRF patients vs. 7/50 minimally/non-displaced DRF patients suffered from osteoporosis at baseline. The diagnosis of osteoporosis was based on the past medical history of the patient including the list of medications (Table 1). Thus, osteoporosis present at baseline does not offer an explanation for the observed differences. Olech et al. reported increased grip strength and range of motion when casting DRFs in elderly patients for 6 weeks compared with 4 weeks. In the present study, we followed the Danish standard of 5 weeks of casting [27]. The optimal duration of casting DRFs in the elderly population remains to be investigated.

The functional status and individual demands of patients should always be taken into consideration when counseling patients about treatment options. Based on our own VOLCON RCT, other RCTs for displaced DRFs in this age group and the results of the present study, we think that non-operative treatment should be the gold standard [6,9,28,29,30,31]. Conversely to the current practice in Denmark, surgeons should clearly state the reasons for opting for surgical treatment of a displaced DRF specifically for patients who are 65 years old or more. For instance, patients depending on walking aids may benefit from operative treatment as the increased stability may allow earlier mobilization and use of walking aids, while casting for 5 weeks may cause not only an immobilization of the wrist, but may also restrict walking. Likewise, patients with bilateral injuries or contralateral functional impairments may be considered for operative treatment in order to facilitate an earlier return to self-care and daily activities [4,7,9]. In such cases, early mobilization may counteract the temporary or permanent loss of independence [32].

When opting for surgery of a displaced DRF in the elderly, one should remember to inform the patients that surgery entails risks besides the surgical complications. Many patients with DRFs are considered frail, which has been found to be a predictor of postoperative morbidity and mortality in patients undergoing surgery in general anesthesia [33]. Furthermore, cognitive impairment is not rare after surgery in the elderly. Steinmetz et al. suggested that inadequate recovery increases the risk of post-operative delirium, which increases the risk of long-term cognitive dysfunctions such as dementia [34].

Another important aspect concerning whether to undergo surgery or not is the patient’s own involvement in the decision making of the treatment. We experienced that a large number of patients who asked to be a part of our primary RCT study did not want to participate due to the fact that they did not want surgery at any cost. The literature shows that public and patient involvement is of great value and importance [35]. For future studies, public and patient involvement should be mandatory in setting up a study such as this.

A limitation of the present study was that the patients ideally should have been blinded. As both groups were non-operatively treated, this would have been feasible if the study was not part of the VOLCON RCT [9,12]. Here, patients in the displaced DRF group had been informed about the radiological severity/displacement of the fracture—blinding of both groups was, thus, not possible in the present study.

Moreover, the number of patients in the two study cohorts limited the study power and the possibility to design the study based on a power analysis. This meant that the difference in complications rates found of 10% might be clinically relevant but more included patients would be needed to demonstrate a statistically significant difference. Furthermore, the external validity, i.e., generalizability, may have been hampered by the fact that more than 90% of the patients were retired. One should, therefore, be careful to draw conclusions for elderly people who are still working.

## 5. Conclusions

In conclusion, in patients above 65 years of age, non-operative treatment, i.e., closed reduction and dorsal casting for 5 weeks, yielded similar complication rates and functional outcomes after 1 year regardless of whether the initial fracture was non-displaced/minimally displaced or still displaced after closed reduction. While initially a closed reduction should still be attempted in order to restore the anatomy, failure to achieve the stipulated radiological criteria may not be as important as we thought in terms of complications and functional outcome. A non-operative treatment strategy for displaced DRFs appears to be a safe and reliable treatment option. The results of our study can inform patients about the expected complications and functional outcomes after dorsal plaster casting for 5 weeks of non-displaced as well as still-displaced DRFs after closed reduction.

## Figures and Tables

**Table 1:** Demographics.

		Displaced	Minimally/Non-Displaced
		n = 50	n = 50
Sex			
	Female	40 (80%)	41 (82%)
	Male	10 (20%)	9 (18%)
Age (years)			
	Median (Min., IQR, Max.)	74 (65, 69–81, 91)	73 (65, 70–78, 100)
AO/OTA classification			
	A1/A2/A3	0/22/16	0/17/15
	B1/B2/B3	0/6/6	2/4/4
	C1/C2/C3	0/5/1	0/2/0
Fractured side			
	Right	24 (48%)	18 (36%)
	Left	26 (52%)	32 (64%)
Hand dominance			
	Right	46 (92%)	43 (86%)
	Left	1 (2%)	4 (8%)
	Ambidextrous	1 (2%)	3 (6%)
	Missing data	2 (4%)	0 (0%)
	Dominant side fractured *	23 (46%)	20 (40%)
Working status			
	Full-time/part-time work	1 (2%)	0 (0%)
	Volunteer work	2 (4%)	3 (6%)
	Retired	45 (90%)	47 (94%)
	Missing data	2 (4%)	0 (0%)
Smoking status			
	Non-smoker	37 (74%)	41 (82%)
	Smoker	9 (18%)	9 (18%)
	Missing data	4 (8%)	0 (0%)
Alcohol consumption **			
	<7/14 units/week	38 (76%)	44 (88%)
	>7/14 units/week	8 (16%)	6 (12%)
	Missing data	4 (8%)	0 (0%)
ASA			
	ASA class 1	13 (26%)	16 (32%)
	ASA class 2	30 (60%)	25 (50%)
	ASA class 3	6 (12%)	9 (18%)
	ASA class 4–5	0 (0%)	0 (0%)
	Missing data	1 (2%)	0 (0%)
Comorbidities			
	Hypertension	23 (46%)	22 (44%)
	Diabetes	6 (12%)	3 (6%)
	Osteoporosis	3 (6%)	7 (14%)
	Depression	4 (8%)	9 (18%)
Medications			
	0	8 (16%)	5 (10%)
	1–4	26 (52%)	38 (76%)
	≥5	16 (32%)	7 (14%)

* A fracture in an ambidextrous patient was not considered a fracture of the dominant side. ** Threshold defined as 7 units/week for females and 14 units/week for males.

## Data Availability

Data may be requested from the corresponding author.

## References

[1] Raudasoja L., Aspinen S., Vastamäki H., Ryhänen J., Hulkkonen S. (2022). Epidemiology and Treatment of Distal Radius Fractures in Finland-a Nationwide Register Study. J. Clin. Med..

[2] Driessen J.H.M., Hansen L., Eriksen S.A., van Onzenoort H.A.W., Henry R.M.A., van den Bergh J., Abrahamsen B., Vestergaard P., de Vries F. (2016). The Epidemiology of Fractures in Denmark in 2011. Osteoporos. Int..

[3] Sundhedsstyrelsen National Klinisk Retningslinje for Behandling Af Håndledsnære Brud (Distal Radiusfraktur). https://www.sst.dk/-/media/Udgivelser/2014/NKR-H%C3%A5ndledsn%C3%A6re-underarmsbrud/National-klinisk-retningslinie-for-behandling-af-haandledsnaere-brud.ashx.

[4] Li Q., Ke C., Han S., Xu X., Cong Y.X., Shang K., Liang J.D., Zhang B.F. (2020). Nonoperative Treatment Versus Volar Locking Plate Fixation for Elderly Patients with Distal Radial Fracture: A Systematic Review and Meta-Analysis. J. Orthop. Surg. Res..

[5] Chen Y., Chen X., Li L., Yan H., Zhou F., Gao W. (2016). Safety and Efficacy of Operative Versus Nonsurgical Management of Distal Radius Fractures in Elderly Patients: A Systematic Review and Meta-Analysis. J. Hand Surg. Am..

[6] Woolnough T., Axelrod D., Bozzo A., Koziarz A., Koziarz F., Oitment C., Gyemi L., Gormley J., Gouveia K., Johal H. (2021). What is the relative effectiveness of the various surgical treatment options for distal radius fractures? A systematic review and network meta-analysis of randomized controlled trials. Clin. Orthop. Relat. Res..

[7] Stephens A.R., Presson A.P., McFarland M.M., Zhang C., Sirniö K., Mulders M.A.M., Schep N.W.L., Tyser A.R., Kazmers N.H. (2020). Volar Locked Plating Versus Closed Reduction and Casting for Acute, Displaced Distal Radial Fractures in the Elderly: A Systematic Review and Meta-Analysis of Randomized Controlled Trials. J. Bone Jt. Surg..

[8] Lawson A., Na M., Naylor J.M., Lewin A.M., Harris I.A. (2021). Volar Locking Plate Fixation Versus Closed Reduction for Distal Radial Fractures in Adults: A Systematic Review and Meta-Analysis. JBJS Rev..

[9] Thorninger R., Wæver D., Tjørnild M., Lind M., Rölfing J.D. (2022). VOLCON: A Randomized Controlled Trial Investigating Complications and Functional Outcome of Volar Plating vs. Casting of Unstable Distal Radius Fractures in Patients Older than 65 Years. J. Orthop. Traumatol..

[10] American Academy of Orthopaedic Surgeons (AAOS) Management of Distal Radius Fractures Evidence-Based Clinical Practice Guideline. www.aaos.org/drfcpg.

[11] The British Society for Surgery of the Hand Best Practice for Management of Distal Radial Fractures. https://www.bssh.ac.uk/_userfiles/pages/files/professionals/Radius/Blue%20Book%20DRF%20Final%20Document.pdf.

[12] Pedersen J., Mortensen S.O., Rölfing J.D., Thorninger R. (2019). A Protocol for a Single-Center, Single-Blinded Randomized-Controlled Trial Investigating Volar Plating Versus Conservative Treatment of Unstable Distal Radius Fractures in Patients Older Than 65 Years. BMC Musculoskelet. Disord..

[13] Schønnemann J.O., Eggers J. (2016). Validation of the Danish Version of the Quick-Disabilities of Arm, Shoulder and Hand Questionnaire. Dan. Med. J..

[14] London D.A., Stepan J.G., Boyer M.I., Calfee R.P. (2014). Performance Characteristics of the Verbal QuickDASH. J. Hand Surg. Am..

[15] Goldhahn J., Beaton D., Ladd A., Macdermid J., Hoang-Kim A. (2014). Recommendation for Measuring Clinical Outcome in Distal Radius Fractures: A Core Set of Domains for Standardized Reporting in Clinical Practice and Research. Arch. Orthop. Trauma Surg..

[16] Franchignoni F., Vercelli S., Giordano A., Sartorio F., Bravini E., Ferriero G. (2014). Minimal clinically important difference of the disabilities of the arm, shoulder and hand outcome measure (DASH) and its shortened version (QuickDASH). J. Orthop. Sport. Phys. Ther..

[17] Institute for Work & Health The Dash Outcome Measure—Frequently Asked Questions (FAQ): What Is Considered to Be a Clinically Important Change for the Dash/QuickDASH?. https://dash.iwh.on.ca/faq.

[18] Hansen A.Ø., Knygsand-Roenhoej K., Ardensø K. (2019). Danish Version of the Patient-Rated Wrist/Hand Evaluation Questionnaire: Translation, Cross-Cultural Adaptation, Test–Retest Reliability and Construct Validity. Hand Ther..

[19] Walenkamp M.M., de Muinck Keizer R.J., Goslings J.C., Vos L.M., Rosenwasser M.P., Schep N.W. (2015). The Minimum Clinically Important Difference of the Patient-Rated Wrist Evaluation Score for Patients with Distal Radius Fractures. Clin. Orthop. Relat. Res..

[20] Saving J., Wahlgren S.S., Olsson K., Enocson A., Ponzer S., Sköldenberg O., Wilcke M., Navarro C.M. (2019). Nonoperative Treatment Compared with Volar Locking Plate Fixation for Dorsally Displaced Distal Radial Fractures in the Elderly: A Randomized Controlled Trial. J. Bone Jt. Surg. Am..

[21] Hamilton G.F., McDonald C., Chenier T.C. (1992). Measurement of Grip Strength: Validity and Reliability of the Sphygmomanometer and Jamar Grip Dynamometer. J. Orthop. Sport. Phys. Ther..

[22] Kim J.K., Park M.G., Shin S.J. (2014). What Is the Minimum Clinically Important Difference in Grip Strength?. Clin. Orthop. Relat. Res..

[23] Cooke M.E., Gu A., Wessel L.E., Koo A., Osei D.A., Fufa D.T. (2022). Incidence of Carpaæ Tunnel Syndrome after Distal Radius Fracture. J. Hand Surg. Glob. Online.

[24] Arora R., Lutz M., Deml C., Krappinger D., Haug L., Gabl M. (2011). A prospective randomized trial comparing nonoperative treatment with volar locking plate fixation for displaced and unstable distal radial fractures in patients sixty-five years of age and older. J. Bone Jt. Surg. Am..

[25] Hassellund S.S., Williksen J.H., Laane M.M., Pripp A., Rosales C.P., Karlsen Ø., Madsen J.E., Frihagen F. (2021). Cast immobilization is non-inferior to volar locking plates in relation to QuickDASH after one year in patients aged 65 years and older: A randomized controlled trial of displaced distal radius fractures. Bone Joint J..

[26] Lawson A., Naylor J.M., Buchbinder R., Ivers R., Balogh Z.J., Smith P., Xuan W., Howard K., Vafa A., Perriman D. (2021). Surgical plating vs closed reduction for fractures in the distal radius in older patients: A randomized clinical trial. JAMA Surg..

[27] Ochen Y., Peek J., van der Velde D., Beeres F.J.P., van Heijl M., Groenwold R.H.H., Houwert R.M., Heng M. (2020). Operative vs nonoperative treatment of distal radius fractures in adults: A systematic review and meta-analysis. JAMA Netw. Open.

[28] Thorninger R., Wæver D., Pedersen J., Tvedegaard-Christensen J., Tjørnild M., Lind M., Rölfing J.D. (2021). Objective Outcome Measures Continue to Improve from 6 to 12 Months after Conservatively Treated Distal Radius Fractures in the Elderly-a Prospective Evaluation of 50 Patients. J. Clin. Med..

[29] Zadeh S.S.T., Moazzeni S.S., Asgari S., Mirbolouk M., Azizi F., Hadaegh F. (2022). Association between Wrist Circumference and Risk of Any Fracture in Adults: Findings from 15 Years of Follow-up in the Tehran Lipid and Glucose Study. J. Clin. Med..

[30] Oh C.H., Kim J., Kim J., Yoon S., Jung Y., Lee H.I., Choi J., Lee S., Han S. (2022). The Association of Low Skeletal Muscle Mass with Complex Distal Radius Fracture. J. Clin. Med..

[31] Olech J., Konieczny G., Tomczyk L., Morasiewicz P. (2021). A Randomized Trial Assessing the Muscle Strength and Range of Motion in Elderly Patients Following Distal Radius Fractures Treated with 4- and 6-Week Cast Immobilization. J. Clin. Med..

[32] Bruyere A., Vernet P., Botero S.S., Igeta Y., Hidalgo Diaz J.J., Liverneaux P. (2018). Conservative Treatment of Distal Fractures after the Age of 65: A Review of Literature. Eur. J. Orthop. Surg. Traumatol..

[33] Shem Tov L., Matot I. (2017). Frailty and Anesthesia. Curr. Opin. Anaesthesiol..

[34] Steinmetz J., Rasmussen L.S. (2018). Anesthesia and the Risk of Dementia in the Elderly. La Presse Méd..

[35] Moss N., Bueno-Cavanillas A., Cano-Ibáñez N., Khan K.S. (2022). Evidence-Based Medicine Needs Patient and Public Involvement to Remain Relevant: A Proposal for a New Curriculum. Semergen.

